# Virulence regulation of Zn^2+^ uptake system *znuABC* on mesophilic *Aeromonas salmonicida* SRW-OG1

**DOI:** 10.3389/fvets.2023.1172123

**Published:** 2023-03-29

**Authors:** Jiajia Wang, Lijun Xiu, Ying Qiao, Youyu Zhang

**Affiliations:** ^1^Fisheries College, Key Laboratory of Healthy Mariculture for the East China Sea, Ministry of Agriculture, Jimei University, Xiamen, Fujian, China; ^2^Fourth Institute of Oceanography, Key Laboratory of Tropical Marine Ecosystem and Bioresource, Ministry of Natural Resources, Beihai, China; ^3^Institute of Electromagnetics and Acoustics, School of Electronic Science and Engineering, Xiamen University, Xiamen, China

**Keywords:** *Aeromonas salmonicida*, *Epinephelus coioides*, *znuABC*, virulence regulation, zinc

## Abstract

Psychrophilic *Aeromonas salmonicida* could not grow above 25°C and therefore thought unable to infect mammals and humans. In our previous study, a mesophilic *A. salmonicida* SRW-OG1 was isolated from *Epinephelus coioides* with furunculosis. Through the analysis of preliminary RNA-seq, it was found that the Zn^2+^ uptake related genes *znuA, znuB* and *znuC* might be involved in the virulence regulation of *A. salmonicida* SRW-OG1. Therefore, the purpose of this study was to explore the effect of *znuABC* silencing on the virulence regulation of *A. salmonicida* SRW-OG1. The results showed that the growth of the *znuA*-RNAi, *znuB*-RNAi, and *znuC*-RNAi strains was severely restricted under the Fe^2+^ starvation, but surprisingly there was no significant difference under the Zn^2+^ restriction. In the absence of Zn^2+^ and Fe^2+^, the expression level of *znuABC* was significantly increased. The motility, biofilm formation, adhesion and hemolysis of the *znuA*-RNAi, *znuB*-RNAi, and *znuC*-RNAi strains were significantly reduced. We also detected the expression of *znuABC* under different growth periods, temperatures, pH, as well as Cu^2+^ and Pb^2+^ stresses. The results showed that *znuABC* was significantly up-regulated in the logarithmic phase and the decline phase of *A. salmonicida*. Interestingly, the trend of expression levels of the *znuABC* at 18, 28, and 37°C was reversed to another Zn^2+^ uptake related gene *zupT*. Taken together, these indicated that the *znuABC* was necessary for *A. salmonicida* SRW-OG1 pathogenicity and environmental adaptability, and was cross regulated by iron starvation, but it was not irreplaceable for *A. salmonicida* SRW-OG1 Zn^2+^ uptake in the host.

## 1. Introduction

*Aeromonas salmonicida* is a facultative anaerobic Gram-negative bacterium, widely existing in aquatic environments and soils (1), which can cause furunculosis in a variety of marine and freshwater fish, especially salmon (*Salmonidae*) and carp (*Cyprinidae*), as well as rainbow trout (*Onco-rhynchus mykiss*), turbot (*Scophthalmus maximus L*.), carp (*Cyprinus carpio*), goldfish (*Carassius auratus*) and so on (2–5). Since *A. salmonicida* has been proven unable to grow above 25°C, and most of them are isolated from cold water teleost fish (6–8), researchers believe that *A. salmonicida* cannot infect mammals and humans for quite a long time (9, 10). However, we previously isolated a mesophilic pathogenic *A. salmonicida* strain from orange-spotted grouper (*Epinephelus coioides*) with furunculosis and named it as *A. salmonicida* SRW-OG1 (11). Meanwhile, there have been some reported cases of human infection with mesophilic *A. salmonicida*. Based on the whole genome comparison between mesophilic *A. salmonicida* C947 and AJ83 isolated from human patients in Spain (12) and *A. salmonicida* SRW-OG1, we found that the genomes of C947, AJ83 and SRW-OG1 were highly similar and shared the vast majority of pathogen-host interaction factors (13), which indicated the possible pathogenicity of *A. salmonicida* SRW-OG1 to mammals and human.

Among all the essential trace elements in living organisms, iron (Fe) is the first abundant transition metal element, followed by zinc (Zn) (14–16). Zn is present in the vast majority of proteins. In prokaryote organisms, Zn-binding proteins account for about 4–8% of the total protein (17–19). It has been found that at least two zinc transport systems existing in bacterial pathogens (20). The *Znu*-like system, which is homologous to the *znuABC* system of the Zn^2+^ transport system of *Escherichia coli* found in a variety of gram-positive and gram-negative bacteria (21), is currently the most commonly used (22). *znuABC* is a high-affinity Zn^2+^ transporter composed of the soluble periplasmic binding protein ZnuA (Formerly YcdH), the high-permeability transmembrane protein ZnuB (Formerly YceA) and the ATPase ZnuC (Formerly YcdI), and is regulated by Zur (zinc uptake system) (23). *znuABC* belongs to the ABC transporter, which is one of the largest transporter gene families that transport a variety of substrates across the membrane by utilizing ATP energy (24, 25). These transporters have two transmembrane domains (TMDs) and two nucleotide binding domains (NBDs). TMD binds to the transported substrate, and NBD provides the energy needed for transport by binding and hydrolyzing two (and sometimes only one) ATP molecules (26). In Gram-positive and Gram-negative bacteria, Zn^2+^ input through the plasma membrane is primarily facilitated by the ABC family transporter *znuABC* (14, 27). Zinc transporters protein *znuABC* have been shown to be useful for a variety of pathogens to overcome nutritional immunity and calmodulin-mediated zinc chelation (28), such as *Pseudomonas aeruginosa* (29) and *Salmonella typhimurium* (30). Studies have shown that the deletion of the *znuA* of *Salmonella enterica* serovar Typhimurium does not limit the growth of pathogens in the culture medium, but greatly reduces the reproductive capacity in the Zn^2+^-limited culture medium (28). Compared with wild-type strain, the biofilm formation ability, movement ability and bacterial competition ability of the Δ*znuABC* mutant strains of *Chromobacterium violaceum* are weakened to a certain degree (31). *Yersinia ruckeri* Δ*znuABC* mutant strains does not slow down under zinc restriction but is significantly weaker than wild-type strains in bacterial competition (32). According to our previous RNA-seq analysis, *znuABC* might be key virulence genes during *A. salmonicida* SRW-OG1 infection at high temperature. But so far, there are no researches studies about the virulence effect of *znuABC* on *A. salmonicida* and whether it is related in the regulation of anti-nutritional immunity.

Therefore, we hypothesized that *znuABC* might play important roles in the anti-nutritional immunity and environmental adaptability, thus affecting the pathogenicity of mesophilic *A. salmonicida* SRW-OG1. The aim of this study was to investigate: (1) the relationship between the *znuABC* and *A. salmonicida* SRW-OG1 Zn^2+^ acquisition, (2) the sensitivity of the *A. salmonicida* SRW-OG1 *znuABC* to environmental stresses, and (3) the effects of *znuABC* on the virulence regulation of *A. salmonicida* SRW-OG1.

## 2. Materials and methods

### 2.1. Bacterial strain and growth conditions

*A. salmonicida* SRW-OG1 is a mesophilic pathogenic strain we previously isolated from *E. coioides* with furunculosis (11). The strain was regularly grown in Luria-Bertani (LB) medium at 28°C, 220 rpm (33). *E. coli* DH5α was purchased from TransGen Biotech (Beijing, China), and grown in LB medium at 37°C, 220 rpm. DH5α pCM 130 was preserved in our laboratory at −80°C.

### 2.2. Knockdown of *A. salmonicida* SRW-OG1 *znuABC* genes

Knocked-down strains of *A. salmonicida* SRW-OG1 *znuA, znuB* and *znuC* were constructed using RNAi, according to the method described by Bittel et al. (34)and Girard et al. (35, 36) with slight modifications. The sequence of *A. salmonicida* SRW-OG1 *znuA, znuB* and *znuC* genes were introduced into Invitrogen Block-iT^TM^ RNAi Designer (https://rnaidesigner.thermofisher.com/rnaiexpress/design.do) to design shRNA online (Supplementary Table 1). T4 DNA ligase was used to ligate the shRNA with the linear pCM130, and the recombinant pCM130 vectors were first transformed into *E. coli* DH5α by heat shock according to the description of Huang et al. (37), and then extracted and transformed into the *A. salmonicida* SRW-OG1 by electroporation. The *A. salmonicida* SRW-OG1 was then cultured in LB with 100 μg/mL tetracycline (TET) at 28°C overnight. Positive colonies were verified by gene sequencing and the expression level of *znuA, znuB* and *znuC* was detected by qRT-PCR (38).

### 2.3. Growth ability detection in Zn^2+^ restriction environment

*A. salmonicida* SRW-OG1 wild-type strain and *znuA, znuB, znuC*-RNAi strains were cultured overnight at 28 °C, adjusted to OD_600nm_ = 0.2, then diluted 1,000 times in gradient, and then 10 μL of bacterial dilution was mixed with 190 μL of Zn^2+^ chelating Luria-Bertani (LB) medium [adding 2 μM Tetrakis-(2-pyridylmethyl)-ethylenediamine (TPEN)] in each well of 96-well plate, and then incubated at 28°C. The concentration of bacterial solution was measured every hour for a total of 36 h and the values of OD_600nm_ was recorded. From the OD_600nm_ value, growth curves were plotted to compare the growth trends of wild-type, *znuA, znuB* and *znuC*-RNAi strains of *A. salmonicida* SRW-OG1 (39). Six independent biological replicates were performed for each time point. Data were presented as mean ± standard deviation (SD).

### 2.4. Growth ability detection in Fe^2+^ restriction environment

The wild-type and *znuA, znuB, znuC*-RNAi strains of *A. salmonicida* SRW-OG1 were incubated at 28°C at 220 rpm to OD_600nm_ = 0.2. Then, the bacterial suspension was diluted 1,000 times, and 10 μL of bacterial dilution was mixed with 190 μL of Fe^2+^ chelating Luria-Bertani (LB) medium (adding 1 μmol/L 2,2-bipyridine) in each well of 96-well plate, and then incubated at 28°C. OD_600nm_ was then recorded hourly for a total of 36 h to compare changes in growth capacity of wild-type and the knocked-down strains (40). Six independent biological replicates were performed for each time point. Data were presented as mean ± standard deviation (SD).

### 2.5. Biofilm forming ability

Biofilm assays for *A. salmonicida* SRW-OG1 was carried out according to Huang et al. (41, 42) with slight modifications. First, the *znuA, znuB, znuC*-RNAi and wild-type strains of *A. salmonicida* SRW-OG1 were cultured under the same conditions (28°C, 220 rpm) overnight, and the OD_600nm_ was adjusted to 0.2. The mixture of 100 μL of Luria-Bertani (LB) medium and 100 μL of bacterial suspension was incubated in the dark at 28°C for 24 h, washed 3 times with phosphate buffered saline (PBS, pH = 7.4, without Calcium and Magnesium), stained with 200 μL of 0.1 % crystal violet for 15 min, then rinsed with PBS, and air-dried at 25°C. The biofilm was dissolved with 200 μL of ethanoic acid (33 %) and quantified with OD_590nm_. Six independent biological replicates were performed for each strain.

### 2.6. Semi-solid agar plate movement ability

The semi-solid agar method was used to determine the motility of *A. salmonicida* SRW-OG1 (39, 43). Wild-type and *znuA, znuB, znuC*-RNAi strains of *A. salmonicida* SRW-OG1 were grown under the same conditions (28°C, 220 rpm) overnight, and adjusted to OD_600nm_ of 0.3. 1 μL of bacterial suspension was dropped vertically on the center of semi-solid agar plate [Luria-Bertani (LB) + 0.3 % agar]. After incubation at 25°C for 30 min, transfer it to 28°C for 20 h. Colony diameters were measured instrumentally, while three independent biological replicates were performed for each strain.

### 2.7. Adhesion ability *in vitro*

The determination of adhesion ability of wild-type and *znuA, znuB, znuC*-RNAi strains of *A. salmonicida* SRW-OG1 were carried out according to the methods of Huang et al. (37, 44) with slight modifications. Briefly, 30 μL of sterile healthy *E. coioides* epidermal mucus was spread evenly on the glass slides, incubated overnight at 25°C in the dark, subsequently fixed with 4% methanol at 25°C for 30 min. The bacterial suspension was adjusted to OD_600nm_ of 0.4. After that, 200 μL of bacterial suspension was taken to cover the area with mucus evenly, and incubated under dark and damp at 28°C for 2 h, then rinsed with phosphate buffered saline for 3 times, air-dried and fixed again with 4% methanol at 25°C for 30 min. Lastly, the slid glasses were stained with 0.1% crystal violet for 3 min, and observed with optical microscope and imaged with digital camera (magnification, × 1000) (45). The number of bacteria was counted from the image using IPwin software (11).

### 2.8. Hemolytic ability

Changes in hemolytic capacity of *A. salmonicida* SRW-OG1 wild-type, *znuA, znuB*, and *znuC*-RNAi strains were compared according to the methods described by Porto-Fett et al. (46, 47). *A. salmonicida* SRW-OG1 were cultured at 28°C, 220 rpm to the logarithmic growth period. A 6 mm round hole was punctured into the Columbia blood agar plate with an oxford cup, and then 100 μL of bacterial suspension was transferred into the center of the hole (48). Afterwards, the blood agar plates were cultured at 37°C for 12 h with the front side up. Finally, whether there was a hemolytic circle was observed and the diameter of the hemolytic circle was measured (49). Three independent biological replicates were performed for each strain.

### 2.9. The expression levels of *znuABC* under different stresses environment

In order to validate the effect of temperature on the expression of *znuABC*, the bacterial solution was adjusted to OD_600nm_ = 0.3–0.4, and the expression level of *znuABC* was detected by qRT-PCR after being cultured at 18, 28, and 37°C for 2–4 h.

In order to validate the effect of pH on the expression of *znuABC*, the bacterial solution was adjusted to OD_600nm_ = 0.3–0.4, then added 1mol/L HCl or NaOH to LB medium and adjusted the medium as pH = 4, 5, 6, 7, or 8. After this, they were incubated at 28°C for 2–4 h, and the expression level of *znuABC* was detected by qRT-PCR.

In order to validate the effect of Cu^2+^ and Pb^2+^ on the expression of *znuABC*, the bacterial solution was adjusted to OD_600nm_ = 0.3–0.4, and added 30 mmol/L of Cu^2+^ or Pb^2+^ to LB medium and they were incubated at 28°C for 2–4 h. Finally, the expression level of *znuABC* was detected by qRT-PCR.

### 2.10. Total RNA extraction and reverse transcription

Total RNA was extracted using TRIzol reagent (Invitrogen, USA) following the manufacturer's instructions (11, 50). Reverse transcription was performed from 2.0 mg of total RNA using A Reverse Mu-MLV cDNA Synthesis Kit (TransGen Biotech Co., Ltd., Beijing, China) following the manufacturer's instructions (11, 51).

### 2.11. Quantitative real-time polymerase chain reaction (qRT-PCR)

The qRT-PCR was performed using a QuantStudio 6 Flex real-time PCR system (Life Technologies Inc., Carlsbad, CA, USA) with the Power Green qPCR Mix (Dongsheng Biotech Co., Guangzhou, China) (52). The total volume of the reaction was 10 μL, which included 0.25 μL forward primer (10 μM), 0.25 μL reverse primer (10 μM), 0.5 μL template, and 9.0 μL 2×Power Green qPCR Mix. The thermal cycler conditions were 95°C for 2 min, followed by 40 cycles of 95°C for 20 s, 58°C for 20 s, and 72°C for 20 s. All qRT-PCR experiments were performed in triplicate using independent samples. Supplementary Table 2 showed all the primers (designed by Primer 5 software, synthesized by Shanghai Sangon Biotechnology) used in the experiment. The relative expression levels of the *znuA, znuB* and *znuC* genes were calculated using the 2^−ΔΔCt^ calculation and the gene expression levels were normalized to *16S rRNA* and housekeeping gene *gyrB* (51, 53).

### 2.12. Statistical analysis

Data were presented as mean ± standard deviation (SD) (*n* = 3), and analyzed by SPSS 24.0 software (IBM, Armonk, NY, USA). Differences were compared by *t*-test, one-way ANOVA followed by the Dunnett's test. *P* < 0.05 was considered statistically significant (45).

### 2.13. Ethics statement

All animal experiments were carried out strictly under the recommendations in the “Guide for the Care and Use of Laboratory Animals” set by the National Institutes of Health. The animal protocols were approved by the Animal Ethics Committee of Jimei University (Acceptance NO JMULAC201159).

## 3. Result

### 3.1. Construction of *znuA, znuB*, and *znuC*-RNAi strains of *A. salmonicida* SRW-OG1

**Figure 1:**
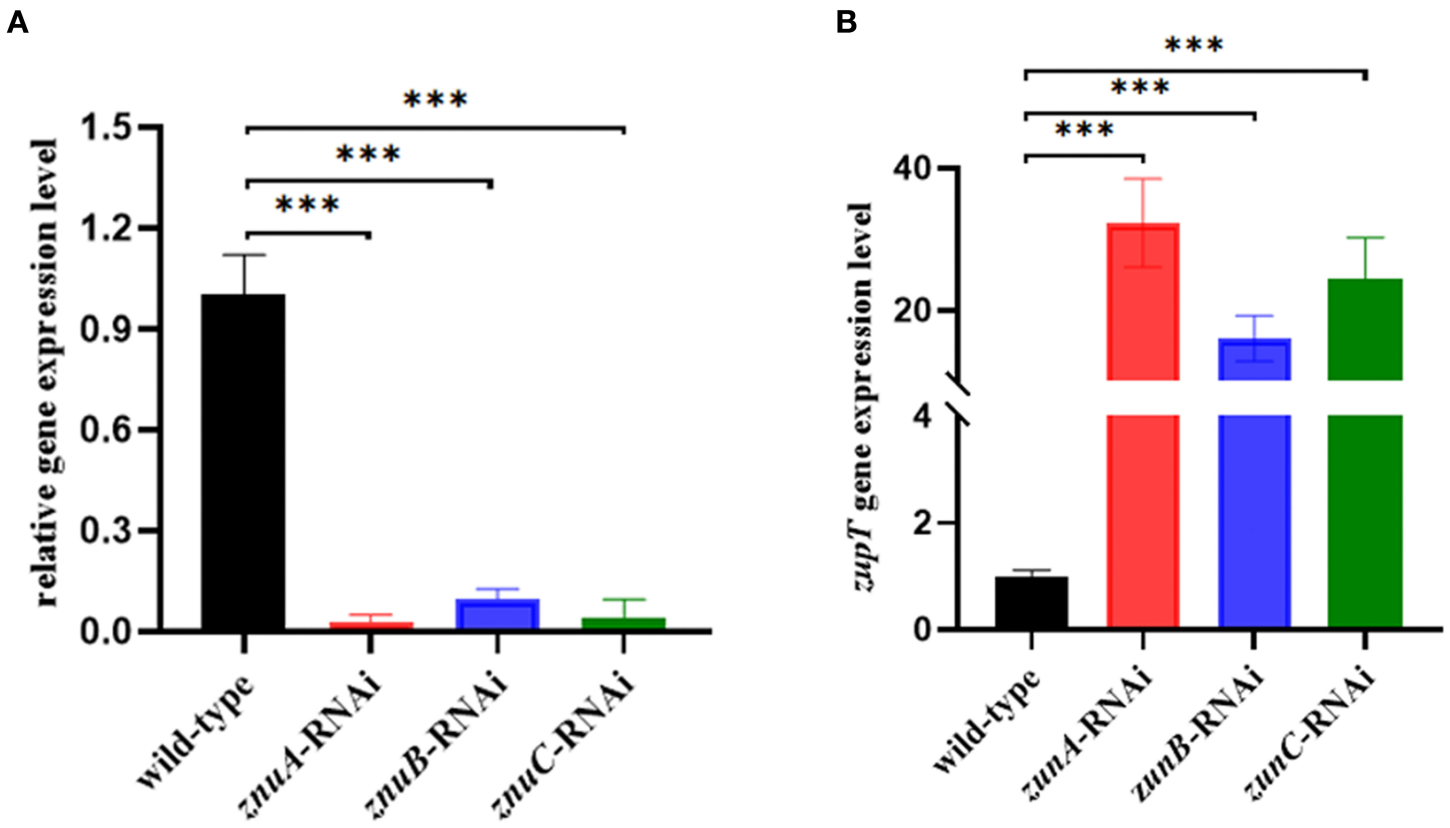
Construction of *A. salmonicida* SRW-OG1 *znuA, znuB*, and *znuC* silencing strains and expression of *zupT* in wild-type and the silencing strains. **(A)** was qRT-RCR validation of silencing efficiency of *znuA*-RNAi, *znuB*-RNAi, and *znuC*-RNAi. **(B)** was the expression levels of *zupT* in wild-type, *znuA, znuB, znuC*-RNAi strains of *A. salmonicida* SRW-OG1; Data were presented as mean ± standard deviation (SD) (*n* = 3). ***P < 0.001.

In the present study, three *znuA, znuB* and *znuC* knocked-down strains were successfully constructed. After the verification by qRT-RCR, the best silencing efficiency was reduced by 97.09, 90.30, and 96.16% (Figure 1A), respectively and was chosen for further research. To verify whether the silencing of *znuA, znuB*, and *znuC* genes affect the function of the low-affinity Zinc-transport system ZupT, the expression levels of *zupT* was determined by qRT-PCR in *A. salmonicida* SRW-OG1 wild-type, *znuA, znuB*, and *znuC*-RNAi strains. The results showed that (Figure 1B) the expression levels of *zupT* in *znuA*-RNAi, *znuB*-RNAi and *znuC*-RNAi were significantly up-regulated by 32.31 times, 16.08 times and 24.33 times, respectively, which indicated that the Zinc-transporter *znuABC* might interact with low-affinity Zinc-transport system ZupT. The increase of *zupT* expression might be a compensation mechanism for *znuABC* silencing.

### 3.2. The effect of *znuA, znuB*, and *znuC* gene on the growth of *A. salmonicida* SRW-OG1 under Zn^2+^ and Fe^2+^ restriction environment

**Figure 2:**
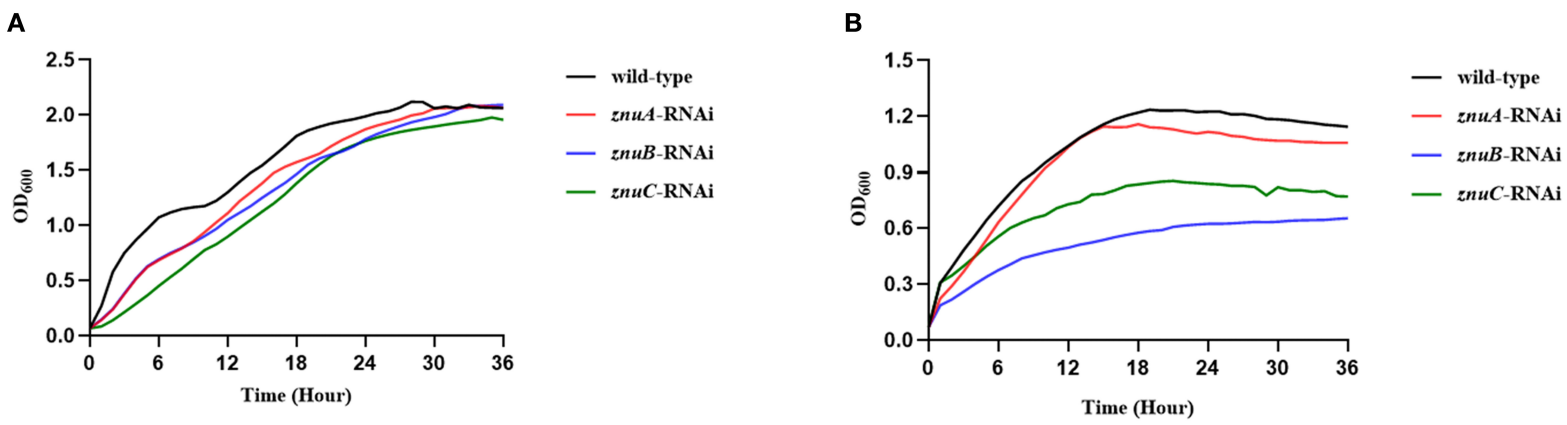
Determination of growth ability in metal ion limited environment. **(A)** was the growth curve under the condition of Zn^2+^ restriction environment. **(B)** was the growth curve under the condition of Fe^2+^ restriction environment.

**Figure 3:**
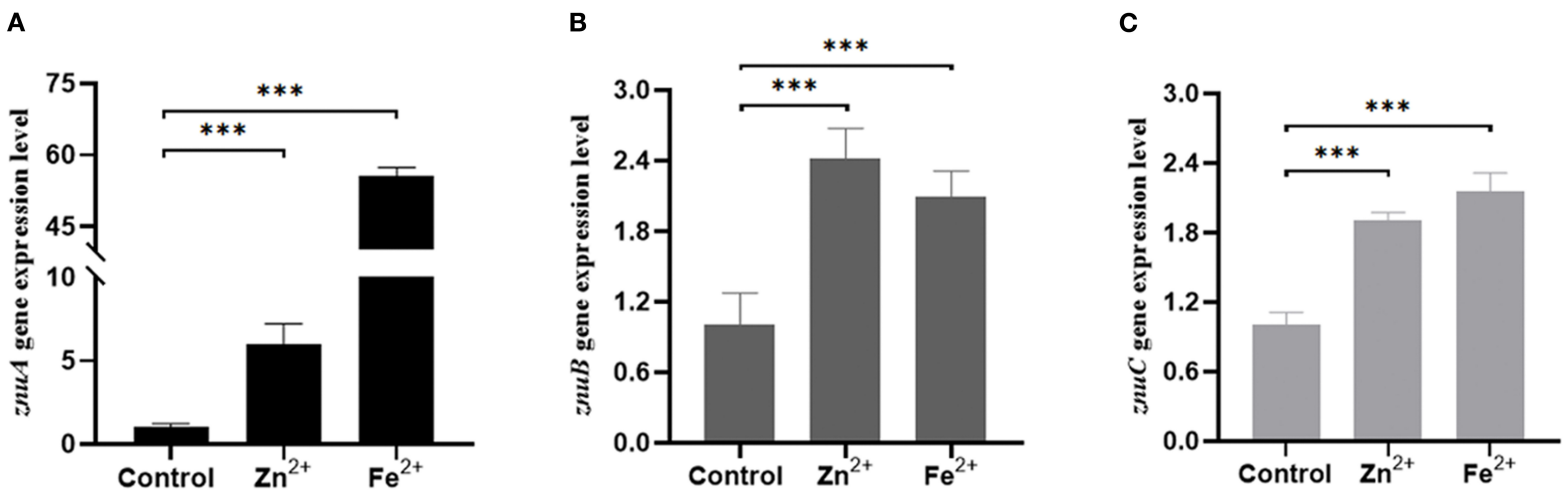
Determination of the expression levels of *znuA, znuB*, and *znuC* in Zn^2+^ and Fe^2+^ limitation. **(A)** was the expression level of *znuA* in Zn^2+^ and Fe^2+^ restriction. **(B)** was the expression level of *znuB* in Zn^2+^ and Fe^2+^ restriction. **(C)** was the expression level of *znuC* in Zn^2+^ and Fe^2+^ restriction. Data were presented as mean ± standard deviation (SD) (*n* = 3). ***P < 0.001.

We determined the growth trend of the *znuA, znuB*, and *znuC* of *A. salmonicida* under Zn^2+^ and Fe^2+^ restriction conditions and the expression levels of *znuA, znuB*, and *znuC* under these limited conditions. In the Zn^2+^ restriction environment, the growth trend of *znuA*-RNAi, *znuB*-RNAi and *znuC*-RNAi were not significantly different than that of the wild-type in the whole growth period (Figure 2A). In the Fe^2+^ restriction environment, the growth trend of *znuA*-RNAi, *znuB*-RNAi and *znuC*-RNAi were significantly weaker than that of the wild-type from logarithmic growth period to decay period (Figure 2B). Among them, under the condition of Fe^2+^ limitation, the growth trends of the three strains declined to different extents, with the reduction of *znuA*-RNAi by 10%, *znuB*-RNAi by 40% and *znuC*-RNAi by 50 %, which might be related to the different functions of the three genes in the transportation process. The expression levels of *znuA, znuB*, and *znuC* in Zn^2+^ and Fe^2+^ limited environment by qRT-PCR (Figure 3). The results revealed that the expression levels of *znuA, znuB* and *znuC* were significantly up-regulated by 6.013, 2.42, and 1.91 times under Zn^2+^ restriction and 55.70, 2.09, and 2.16 times under Fe^2+^ restriction.

### 3.3. The effect of *znuA, znuB, and znuC* on the biofilm forming ability of *A. salmonicida* SRW-OG1

**Figure 4:**
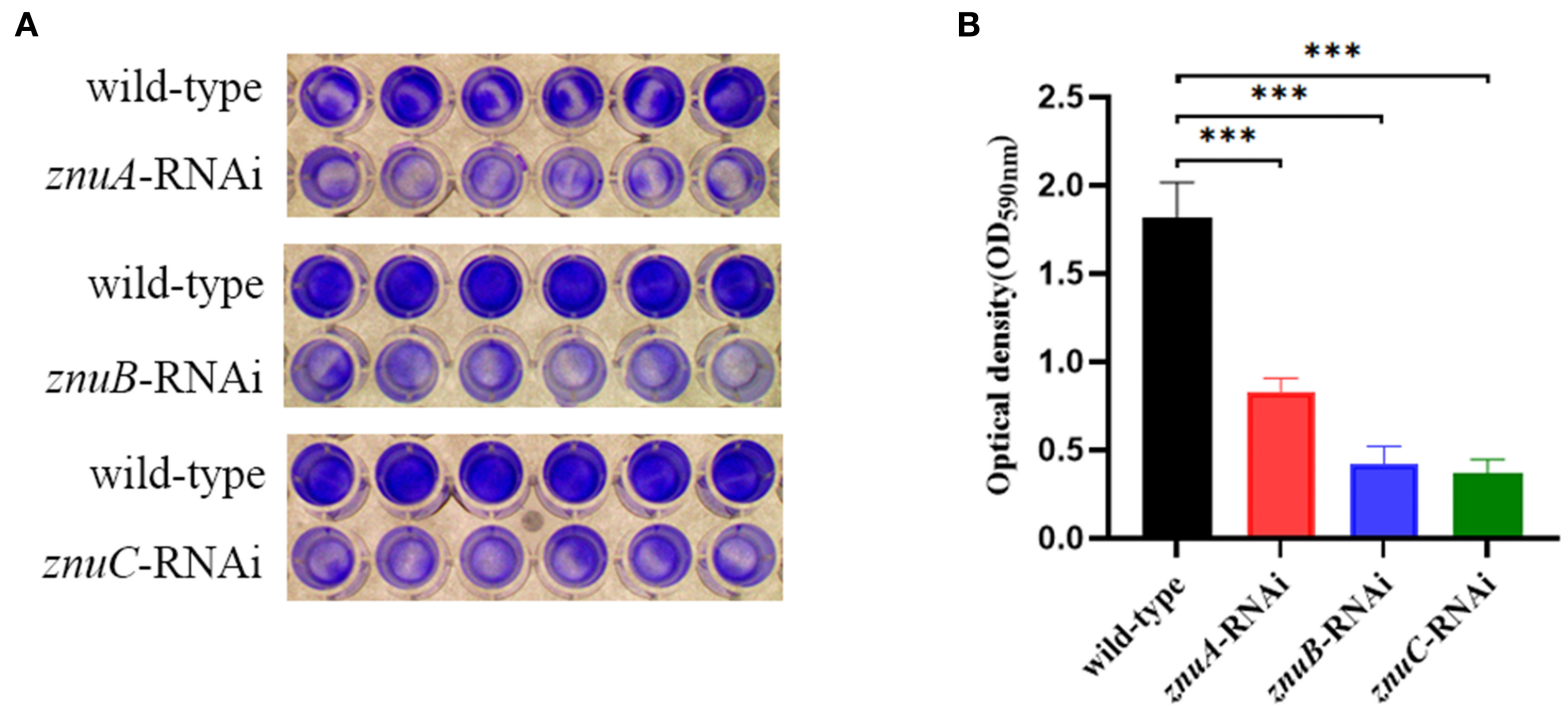
Biofilm forming ability assay of wild-type, *znuA, znuB*, and *znuC*-RNAi strains of *A. salmonicida* SRW-OG1. **(A)** was the typical figure of the biofilm formation of wild-type, *znuA, znuB* and *znuC*-RNAi strains of *A. salmonicida* SRW-OG1; **(B)** was the biofilm formation histogram of wild-type, *znuA, znuB*, and *znuC*-RNAi strains of *A. salmonicida* SRW-OG1. Data were presented as mean ± standard deviation (SD) (*n* = 3). ***P < 0.001.

The capability of biofilm formation was determined by crystal violet staining. Our results showed that the biofilm forming of *znuA*-RNAi, *znuB*-RNAi and *znuC*-RNAi were significantly weaker than that of the wild-type after culturing for 24 h under the same conditions, and the biofilm formation ability decreased by 2.18, 4.30, and 4.93 times, respectively (Figure 4).

### 3.4. The effect of *znuA, znuB*, and *znuC* on motility of *A. salmonicida* SRW-OG1

**Figure 5:**
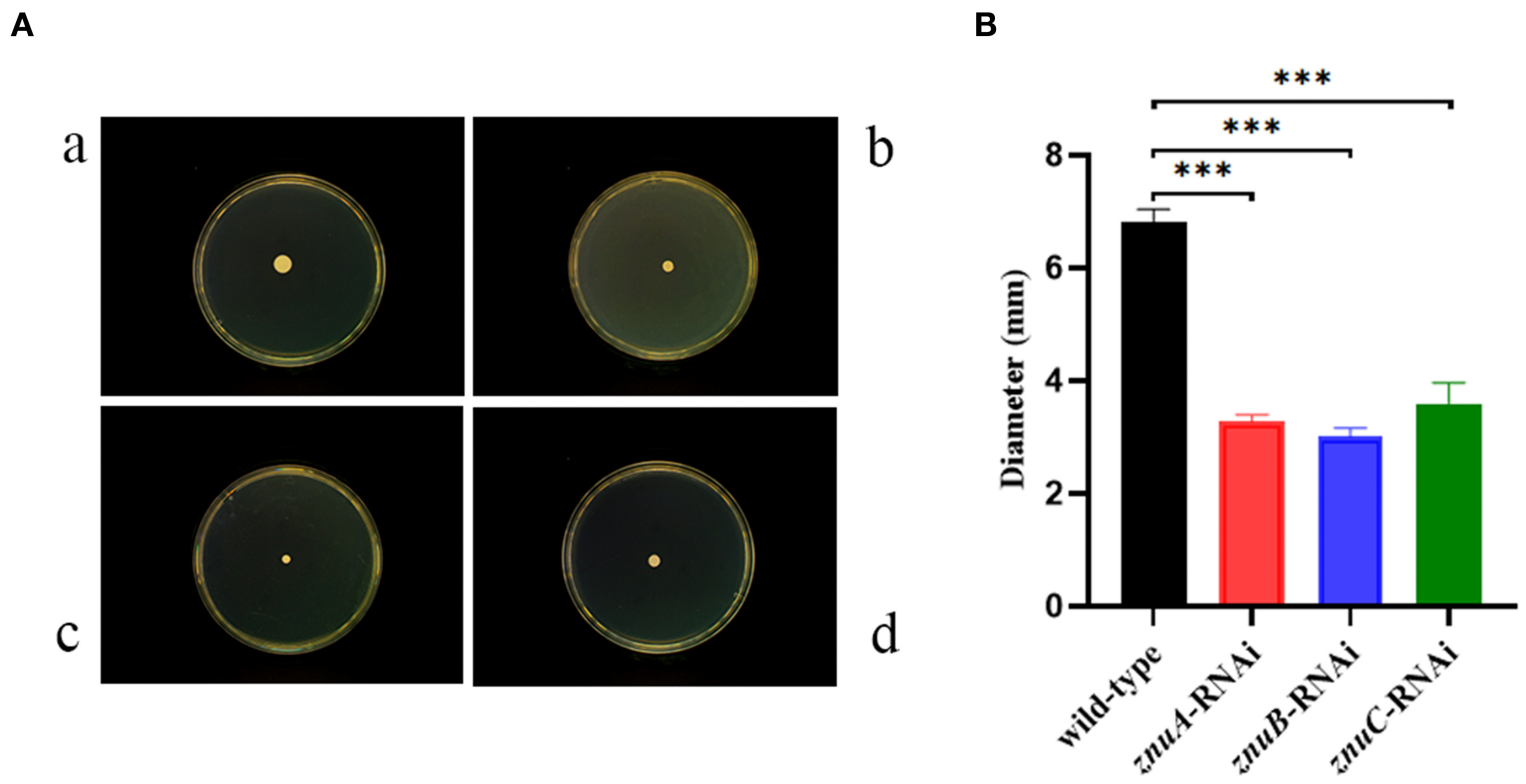
Single colony motility assay of wild-type and *znuA-RNAi, znuB-RNAi, znuC-RNAi* of *A. salmonicida* SRW-OG1. **(A)** was the typical observation of plate movement of the wild-type (a), *znuA*-RNAi (b), *znuB*-RNAi (c) and *znuC*-RNAi (d) strains; **(B)** was the colony diameter histogram. Data were presented as mean ± standard deviation (SD) (*n* = 3). *** *P* < 0.001.

The motility assay showed (Figure 5) that the single colony diameters of wild-type, *znuA, znuB* and *znuC*-RNAi strains of *A. salmonicida* SRW-OG1 placed on semi-solid agar plates for 18 h were about 6.826 ± 0.21 mm, 3.271 ± 0.12 mm, 3.006 ± 0.15 mm and 3.594 ± 0.36 mm, respectively. Statistical analysis revealed a significant 2.09, 2.27, and 2.05 times reduction, respectively in motility of *znuA*-RNAi, *znuB*-RNAi and *znuC*-RNAi compared with wild-type strain. Therefore, it seemed that *znuA, znuB*, and *znuC* were positively related to the motility of *A. salmonicida* SRW-OG1.

### 3.5. The effect of *znuA, znuB*, and *znuC* on hemolytic ability of *A. salmonicida* SRW-OG1

**Figure 6:**
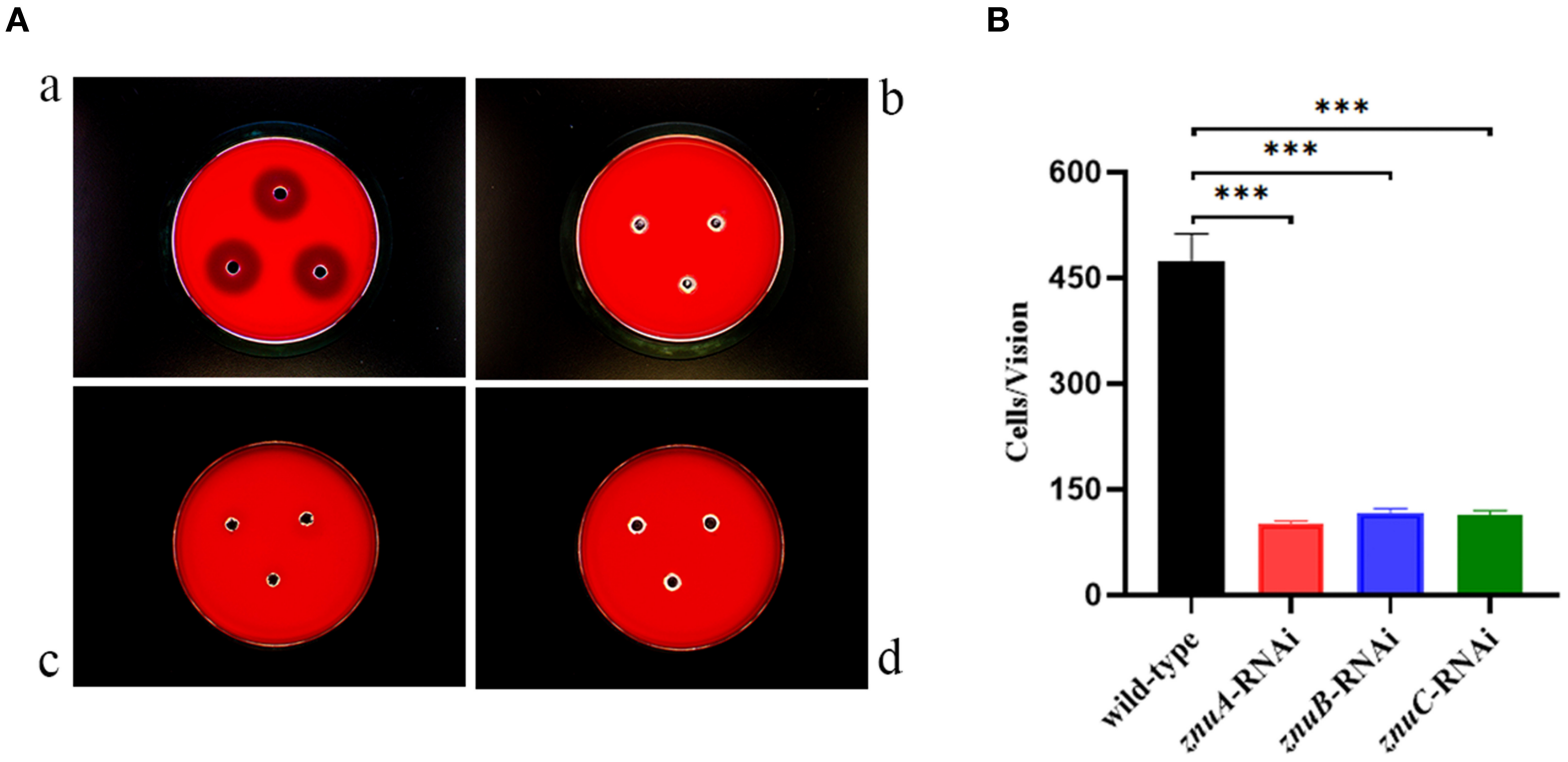
Determination of hemolytic ability and adhesion ability *in vitro* of wild-type, *znuA*-RNAi, *znuB*-RNAi and *znuC*-RNAi strains of *A. salmonicida* SRW-OG1. **(A)** was the typical observation of the hemolytic assay of wild-type (a), *znuA*-RNAi (b), *znuB*-RNAi (c) and *znuC*-RNAi (d) of *A. salmonicida* SRW-OG1 under the optical microscope; **(B)** is the histogram of the number of wild-type, *znuA*-RNAi, *znuB*-RNAi and *znuC*-RNAi strains of *A. salmonicida* SRW-OG1 in the field of optical microscope during *in vitro* adhesion test. Data were presented as mean ± standard deviation (SD) (*n* = 3). *** *P* < 0.001.

According to our recently published study, the *A. salmonicida* SRW-OG1 contains the aerolysin and hemolysin (5). In the present study, the hemolysis activity of *A. salmonicida* SRW-OG1 wild-type, *znuA*-RNAi, *znuB*-RNAi and *znuC*-RNAi strains were compared. As compared with wild-type strain, *znuA*-RNAi, *znuB*-RNAi and *znuC*-RNAi strains have no obvious hemolysis circle, which indicated that the hemolytic capacity of the *znuA*-RNAi, *znuB*-RNAi and *znuC*-RNAi were significantly lower than that of the wild-type (Figure 6A).

### 3.6. The effect of *znuA, znuB*, and *znuC* on adhesion ability of *A. salmonicida* SRW-OG1

As compared with wild-type strain, the adhesion ability of the *znuA*-RNAi, *znuB*-RNAi and *znuC*-RNAi strains *in vitro* decreased by 4.65, 4.06, and 4.16 times, respectively (Figure 6B), which showed that the number of wild-type adhered to the mucus was significantly more than that of *znuA*-RNAi, *znuB*-RNAi and *znuC*-RNAi, and indicated that the adhesion ability *in vitro* of *znuA*-RNAi, *znuB*-RNAi and *znuC*-RNAi were significantly lower than that of wild-type.

### 3.7. The expression levels of *znuA, znuB*, and *znuC* at different growth stages of *A. salmonicida* SRW-OG1

**Figure 7:**
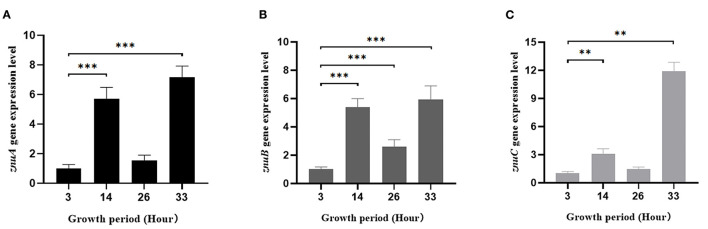
Determination of the expression levels of *znuA, znuB*, and *znuC* in different growth stages. The expression levels of *znuA* **(A)**, *znuB* **(B)** and *znuC* **(C)** in different growth period. Data were presented as mean ± standard deviation (SD) (*n* = 3). **P < 0.01, ***P < 0.001.

We determined the expression level of *znuA, znuB* and *znuC* in different growth periods (Figure 7). In the logarithmic phase (14 h), compared with the growth adaptation phase (3 h), the expression levels of *znuA, znuB* and *znuC* were increased significantly by 5.73, 5.39, and 3.08 times, respectively. At the growth stability stage (26 h), the expression of *znuB* was significantly increased by 2.62 times, while the differences between *znuA* and *znuC* were not statistically significant. During the growth recession (33 h), the expression levels of *znuA, znuB*, and *znuC* were significantly increased by 7.17, 5.93, and 11.92 times, respectively.

### 3.8. Determination of *znuA, znuB*, and *znuC* expression levels under different stress environments

**Figure 8:**
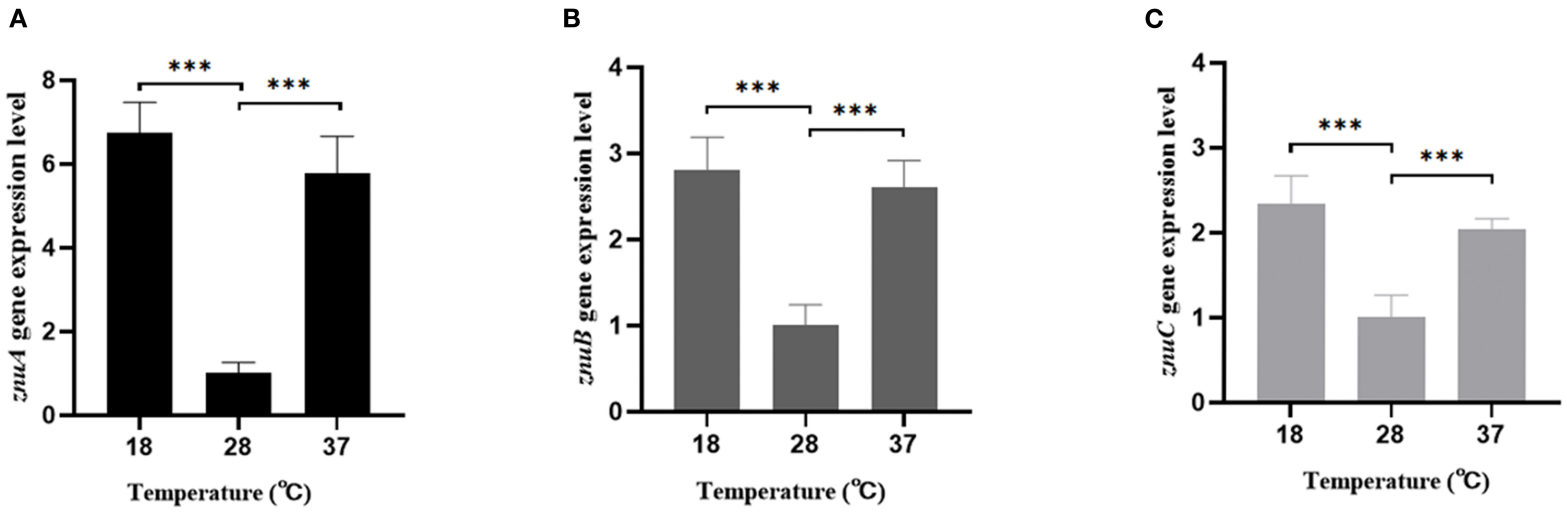
The expression levels of *znuA, znuB*, and *znuC* under different temperature. **(A)** was the expression levels of *znuA* at 18, 28, and 37°C; **(B)** was the expression levels of *znuB* at 18, 28, and 37°C; **(C)** was the expression levels of *znuC* at 18, 28, and 37°C. Data were presented as mean ± standard deviation (SD) (*n* = 3). ***P < 0.001.

According to our previous RNA-seq analysis, compared with the expression levels of *znuA, znuB*, and *znuC* at 18°C, the expression levels at 28°C were down-regulated by 3.85, 3.13 and 2.08 times but there was no significant difference at 37°C. Verified by qRT-PCR, the *znuA, znuB* and *znuC* gene expression levels were consistent with the RNA-seq (Figure 8): compared with the expression levels of *znuA, znuB*, and *znuC* genes at 18°C, the expression level at 28°C was down-regulated by 6.76, 2.81, and 2.34 times but there was no significant difference at 37°C.

**Figure 9:**
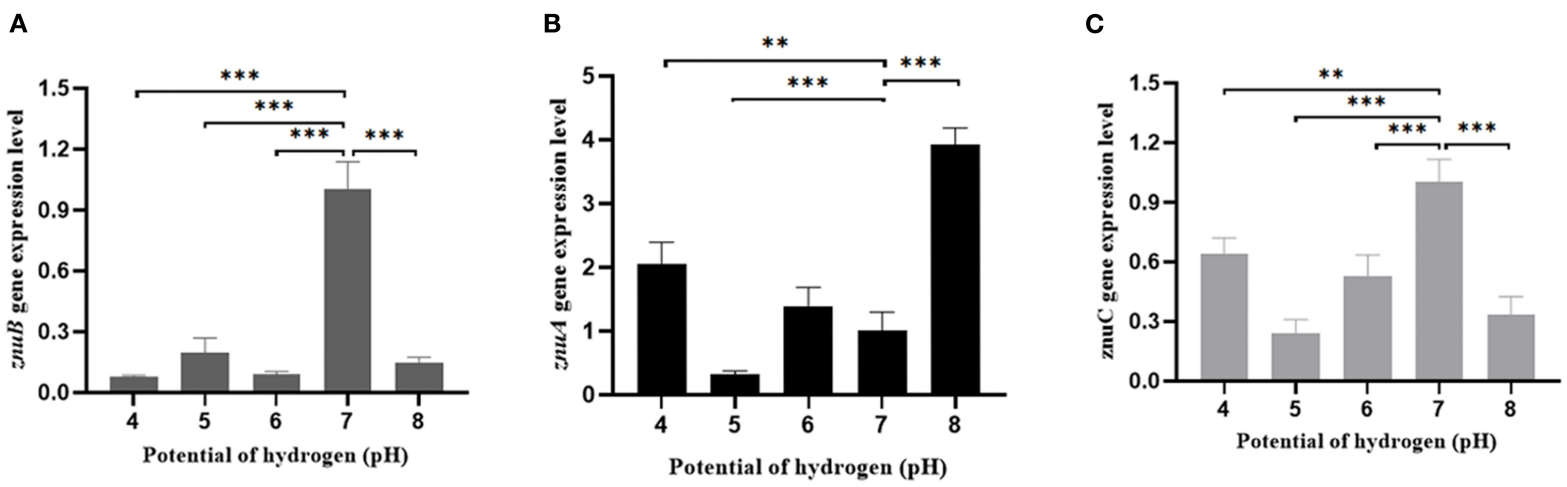
The expression levels of *znuA, znuB* and *znuC* gene under different potential of hydrogen. **(A)** was the expression levels of *znuA* gene at pH = 4, 5, 6, 7 and 8; **(B)** was the expression levels of *znuB* gene at pH = 4, 5, 6, 7 and 8; **(C)** was the expression levels of *znuC* gene at pH = 4, 5, 6, 7, and 8. Data were presented as mean ± standard deviation (SD) (*n* = 3). **P < 0.01, ***P < 0.001.

**Figure 10:**
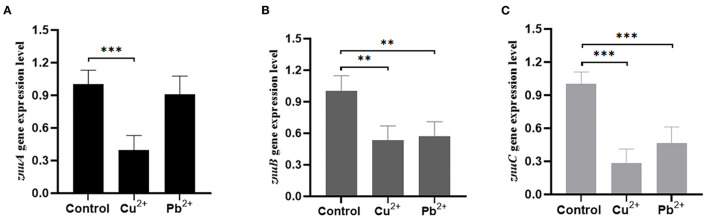
The expression levels of *znuA, znuB* and *znuC* gene under different heavy metal ion stresses. **(A)** was the expression levels of *znuA* under Cu^2+^ and Pb^2+^ stresses; **(B)** was the expression levels of *znuB* under Cu^2+^ and Pb^2+^ stresses; **(C)** was the expression levels of *znuC* under Cu^2+^ and Pb^2+^ stresses. Data were presented as mean ± standard deviation (SD) (*n* = 3). **P < 0.01, ***P < 0.001.

In comparison with the pH = 7 group, the expression level of *znuA* was significantly up-regulated by 2.05 and 3.92 times at pH = 4 and 8, respectively, and it was significantly down-regulated by 3.23 times at pH = 5, although there was a slight increase of *znuA* expression at pH = 6 (Figure 9A). The expression level of *znuB* was significantly down-regulated by 13.07, 5.06, 11.17, and 6.89 times at pH = 4, 5, 6, and 8, respectively (Figure 9B). The expression level of *znuC* was significantly down-regulated by 1.57, 4.16, 1.89, and 2.97 times at pH = 4, 5, 6, and 8, respectively (Figure 9C). The expression of *znuA* decreased by 2.52 times under Cu^2+^ stress, while under Pb^2+^ stress, there was no significant difference (Figure 10A). The expression of *znuB* decreased by 1.87 and 1.77 times under Cu^2+^ and Pb^2+^ stress, respectively (Figure 10B). The expression of *znuC* decreased by 3.53 and 2.15 times under Cu^2+^ and Pb^2+^ stress, respectively (Figure 10C).

## 4. Discussion

Divalent transition metal ions such as Fe, Mn, Zn, Cd, etc., are necessary for all living organisms. During the infection, pathogens must compete with the host for micronutrients in order to survive and proliferate. However, the host utilizes the micronutrient requirement of pathogen to evolve a complex mechanism to battle with them, which known as nutritional immunity. At present, most of the research on nutritional immunity focuses on mammals, but there is little research on nutritional immunity in fish, and research on zinc nutritional immunity in fish is almost non-existent. Although zinc is essential for organisms, excessive amounts of zinc can be harmful to cells (54). At physiological concentrations, zinc may have a protective effect against free radical formation (55, 56), however, excess zinc has been shown to induce cytotoxicity by competing for protein binding with other metals (57), and exposure to high concentrations of zinc has been hypothesized to cause protein degeneration and dysfunction (58). There are mainly two Zinc transporters for zinc-uptake in most gram-negative bacteria, one is the *znuABC*, a high-affinity Zinc transport system, and the other is the low-affinity ZIP family Zn transporter ZupT (59). *znuABC* is composed of soluble periplasmic binding protein ZnuA, high-permeability transmembrane protein ZnuB and ATP enzyme ZnuC, and is regulated by Zur (zinc uptake system) (23). Zur is a member of the Fur-like family of transcriptional regulators with the ability to sense and respond to minute changes in intracellular zinc concentration (60). Zur has a structurally stable zinc binding site (C site) and 1 or 2 binding sites (M and D sites) that regulate DNA binding (61–63). Therefore, there are two situations: under the condition of sufficient zinc, excessive Zn^2+^ interact with M and / or D sites, causing conformational changes, and binding regulatory factors to *znuA* promoter, thus inhibiting its transcription, and in the case of Zn^2+^ deficiency, the M and / or D sites are not occupied, which leads to the instability of Zur, and the regulator cannot inhibit the transcription of *znuA*, thus allowing Zn^2+^ to be absorbed (64, 65).

Studies have shown that the growth of *Salmonella enterica serovar* Typhimurium without *zupT* is not restricted under normal conditions (66), but there is a growth defect under zinc restriction, and that *S*. Typhimurium without *znuABC* is not restricted under zinc restriction (67), which is approximately the same as our results, and that the growth of *A. salmonicida* SRW-OG1 *znuA*-RNAi, *znuB*-RNAi and *znuC*-RNAi silent strains in Zn^2+^ chelating medium is not restricted. However, the growth of *A. salmonicida* SRW-OG1 was significantly inhibited in the Fe^2+^ chelating medium. Therefore, we speculated that the uptake of zinc by the *znuABC* system was limited under the Zn^2+^ limitation condition, but another Zn transport system ZupT played a role to compensate for the *znuABC*. Studies have shown that excessive Zn^2+^ interferes with the steady state of Fe^2+^ in *E. coli*, and excessive Zn^2+^ instantaneously up-regulates the Fe^2+^ absorption genes and down-regulates the Fe^2+^ storage genes, thereby increasing the cellular iron quota (68). According to Huang et al., the *znuA, znuB* and *znuC* of *Pseudomonas plecoglossicida* were transcribed into an mRNA molecule and negatively regulated by the Fur binding box located upstream of *znuC*, and the regulation of Fur on ZnuCBA indicates that there is cross-talk between the acquisition of Zn^2+^ and Fe^2+^ and the nutritional immunity of the host, and it also proves that the separation of iron in the host may occur before the separation of zinc (41). Under the condition of Zn^2+^ and Fe^2+^ restriction, the *znuA, znuB* and *znuC* of *A. salmonicida* SRW-OG1 were significantly up-regulated, which indicated an interaction between Fur and *znuABC* during uptake of zinc and iron by *A. salmonicida*, but whether the transporter *znuABC* of *A. salmonicida* SRW-OG1 was regulated by Fur needed further research and verification.

Bacteria had specific resistance genes against heavy metal ions such as Ag^+^, AsO^3−^, Cd^2+^, Co^2+^, Cu^2+^, Hg^2+^, Ni^2+^, and Pb^2+^, common membrane cation pumps were Cd^2+^-resistance ATPase of Gram-positive bacteria (CadA) and other bacteria, animal and plant P-type ATPases (54). Cd^2+^, Zn^2+^, Co^2+^ and Ni^2+^ are pumped from Gram-bacteria by three polypeptide membrane complex that is NOT an ATPase but functions as a divalent cation/2H^+^ antiporter. The complex consists of an inner membrane protein (CzcA), an outer membrane protein (CzcC) and a protein associated with both membranes (CzcB) (69). The Zur regulon of *E. coli* contains four operons: *znuABC, zinT, ykgMO* and *pliG* (70, 71). Many bacteria such as *Paracoccus denitrificans* (72), *Mycobacterium tuberculosis* (73) and *Bacillus subtilis* (74) contain *znuABC* or Znu-like members of the ABC superfamily. The metal-resistant betaproteobacterium *Cupriavidus metallidurans* had multiple metal resistance systems, including the CzcCBA transmembrane efflux system, which although it does not contain *znuABC* has the ZupT of the ZIP family that maintains zinc homeostasis (75). According to our results, *znuA, znuB*, and *znuC* were all significantly up-regulated to varying degrees under heavy metal ion stresses, which suggested that *znuA, znuB*, and *znuC* might be potential toxic ion resistance genes.

*A. salmonicida* SRW-OG1, isolated from warm water marine fish *E. coioides*, could normally grow and infect at high temperatures (28–37°C). *A. salmonicida* was first isolated from human blood in 2014, before it was determined to infect a variety of freshwater fish but was not pathogenic to mammals and humans because it could not grow at 37°C (76). Cases of human infection by *A. salmonicida* have been reported, such as the first case of endocarditis caused by *A. salmonicida* infection (77). The isolation of C947 strains from patients with acute gastroenteritis and the isolation of AJ83 strains from patients with post-traumatic cellulitis of the foot (12). Based on pathogen-host interaction factor analysis by Qi et al. (13) against *A. salmonicida* SRW-OG1, C947, and AJ83 strains and transcriptome data analysis by Chen et al. (5) on *A. salmonicida* SRW-OG1 at different temperatures, *A. salmonicida* SRW-OG1 has a great potential to infect mammals and humans. Notably, the remarkably high expression of *znuA, znuB*, and *znuC* genes at 18 and 37°C also indicated that they might be related to the pathogenesis under different temperatures. Considering that SRW-OG1 had more than one zinc uptake and transport system, we speculated that the emergence of this U-type expression trend might be an adaptive mechanism for different hosts/environments formed by *A. salmonicida* in the long-term evolution process. At the same time, it also indicates that *A. salmonicida* SRW-OG1 can infect marine and freshwater fish at low temperature (18 and 28°C) and mammals or human at high temperature (37°C).

The biofilm formation ability, adhesion ability, hemolytic activity and motility of *A. salmonicida* SRW-OG1 are the basis for judging the virulence. According to our results, the biofilm formation ability, motility, adhesion and hemolysis ability of *znuA*-RNAi, *znuB*-RNAi and *znuC*-RNAi strains were weakened to different degrees, and these abilities were the basic conditions for bacteria to infect the host or cells. These indicated that *znuA, znuB*, and *znuC* genes were involved in the pathogenic process of *A. salmonicida* SRW-OG1 and played a key role in its survival in adversity.

## 5. Conclusion

Our results showed that *znuA, znuB*, and *znuC* were closely related to the biofilm formation, motility, adhesion and hemolytic activity of *A. salmonicida* SRW-OG1. Meanwhile, the expression of *znuA, znuB*, and *znuC* were significantly affected by the environmental factors such as temperature, pH and heavy metals. Under the Zn^2+^ and Fe^2+^ limitation, the uptake of Zn^2+^ by *znuABC* was limited, but ZupT could compensate for it, and the high expression of *znuA, znuB*, and *znuC* might indicate a cross-talk of Fur and *znuABC*. Therefore, *znuA, znuB*, and *znuC* appears to be vital for the general fitness of *A. salmonicida* SRW-OG1, so as to indirectly affect its efficiency of infecting the host.

## Data availability statement

The original contributions presented in the study are included in the article/Supplementary material, further inquiries can be directed to the corresponding author.

## Ethics statement

All animal experiments were carried out strictly under the recommendations in the Guide for the Care and Use of Laboratory Animals set by the National Institutes of Health. The animal protocols were approved by the Animal Ethics Committee of Jimei University (Acceptance NO JMULAC201159).

## Author contributions

YQ and YZ conceived the experiments. JW, LX, and YQ conducted the experiments. JW and YZ wrote the manuscript. All authors assisted in the collection and interpretation of data.
