# Supplementary material for: Virulence regulation of Zn2+ uptake system znuABC on mesophilic Aeromonas salmonicida SRW-OG1
Source: Front Vet Sci. 2023 Mar 29;10:1172123. doi: 10.3389/fvets.2023.1172123 (PMC10090552; doi:10.3389/fvets.2023.1172123)
Supplement: Supplementary file 1 [file Data_Sheet_1.docx]

Table. S1 The sequences of shRNA for *znuA*, *znuB* and *znuC*

| Name | Sequence |
| --- | --- |
| *znuA*-RNAi  *znuB*-RNAi  *znuC*-RNAi | F: 5′-TGC AAT GGC AAA CAC GAA CAG CTT CAA GAG AGC TGT TCG TGT TTG CCA TTG CTT TTT TT-3′  R: 5'-GTA CAA AAA AAG CGA ATT CTT ACA CTG ATC ACT CTC TTG AAG TGA TCA GTG TAA GAA TTC GCA TGC A-3′  F:5'-TGC TCA GCC TCT CTT GGT ATC ATT CAA GAG ATG ATA CCA AGA GAG GCT GAG CTT TTT TT-3'  R:5'-GTA CAA AAA AAG CTC AGC CTC TCT TGG TATC ATC TCT TGA ATG ATA CCA AGA GAG GCT GAG CAT GCA-3'  F:5'-TGG TCT ACA CCC ACC ATC ATC ATT CAA GAG ATG ATG ATG GTG GGT GTA GAC CTT TTT TT-3'  R:5'-GTA CAA AAA AAG GTC TAC ACC CAC CAT CAT CAT CTC TTG AAT GAT GAT GGT GGG TGT AGA CCA TGC A-3' |

Table. S2 Primers for qRT-PCR

| Gene Name | Primers |
| --- | --- |
| *16S-rRNA* | F: 5′-GTTGGGAGGAAGGGCAGTAAG-3′  R: 5′-ATCTAGGCATTTCACCGCTACA-3′ |
| *znuA* | F: 5′-GCGGTGAGTCTGCCTGTC-3′  R: 5'-TTGGCCATGAAACCTTCC-3′ |
| *znuB* | F: 5′-GCGTACTGATGCTGCTGATTG-3′  R: 5'-CAGGCTTTCTTGAACTGGCTC-3′ |
| *znuC* | F: 5′-TGAGGTGATCTGTCTCAATCGTC-3′  R: 5'-TATTGATTGCGAGACGGCAG-3′ |
| *gyrB* | F: 5′-CGTGGGCGTCTCAGTGGTT-3′  R: 5'-CAGGGTATCGGTAAAGATGGTC-3′ |
| *zupT* | F: 5′-TGGGTTGCGGTATTGGTCG-3′  R: 5′-AGAGTGGCGTGGGTCTGGTG-3′ |
